# Predictors Linking Obesity and the Gut Microbiome (the PROMISE Study): Protocol and Recruitment Strategy for a Cross-Sectional Study on Pathways That Affect the Gut Microbiome and Its Impact on Obesity

**DOI:** 10.2196/14529

**Published:** 2019-08-26

**Authors:** Sophie Kindleysides, Rozanne Kruger, Jeroen Douwes, Gerald W Tannock, Nikki Renall, Joanne Slater, Blair Lawley, Anne-Thea McGill, Niamh Brennan, Moana Manukia, Marilize Richter, Ridvan Tupai-Firestone, T Leigh Signal, Philippa Gander, Stephen R Stannard, Bernhard H Breier

**Affiliations:** 1 School of Exercise, Sport and Nutrition College of Health Massey University Auckland New Zealand; 2 Centre for Public Health Research Massey University Wellington New Zealand; 3 Department of Microbiology and Immunology University of Otago Dunedin New Zealand; 4 Microbiome Otago University of Otago Dunedin New Zealand; 5 Riddet Centre of Research Excellence Palmerston North New Zealand; 6 School of Health & Human Sciences Southern Cross University East Lismore Australia; 7 The Fono Health and Social Services Auckland New Zealand; 8 Sleep/Wake Research Centre College of Health Massey University Wellington New Zealand

**Keywords:** diet, gut microbiome, body composition, women, overweight, obesity, physical activity, taste perception, sleep, metabolic diseases

## Abstract

**Background:**

The prevalence of obesity has increased substantially over recent decades and is associated with considerable health inequalities. Although the causes of obesity are complex, key drivers include overconsumption of highly palatable, energy-dense, and nutrient-poor foods, which have a profound impact on the composition and function of the gut microbiome. Alterations to the microbiome may play a critical role in obesity by affecting energy extraction from food and subsequent energy metabolism and fat storage.

**Objective:**

We report the study protocol and recruitment strategy of the PRedictors linking Obesity and the gut MIcrobiomE (PROMISE) study, which characterizes the gut microbiome in 2 populations with different metabolic disease risk (Pacific and European women) and different body fat profiles (normal and obese). It investigates (1) the role of gut microbiome composition and functionality in obesity and (2) the interactions between dietary intake; eating behavior; sweet, fat, and bitter taste perception; and sleep and physical activity; and their impact on the gut microbiome, metabolic and endocrine regulation, and body fat profiles.

**Methods:**

Healthy Pacific and New Zealand (NZ) European women aged between 18 and 45 years from the Auckland region were recruited for this cross-sectional study. Participants were recruited such that half in each group had either a normal weight (body mass index [BMI] 18.5-24.9 kg/m^2^) or were obese (BMI ≥30.0 kg/m^2^). In addition to anthropometric measurements and assessment of the body fat content using dual-energy x-ray absorptiometry, participants completed sweet, fat, and bitter taste perception tests; food records; and sleep diaries; and they wore accelerometers to assess physical activity and sleep. Fasting blood samples were analyzed for metabolic and endocrine biomarkers and DNA extracted from fecal samples was analyzed by shotgun sequencing. Participants completed questionnaires on dietary intake, eating behavior, sleep, and physical activity. Data were analyzed using descriptive and multivariate regression methods to assess the associations between dietary intake, taste perception, sleep, physical activity, gut microbiome complexity and functionality, and host metabolic and body fat profiles.

**Results:**

Of the initial 351 women enrolled, 142 Pacific women and 162 NZ European women completed the study protocol. A partnership with a Pacific primary health and social services provider facilitated the recruitment of Pacific women, involving direct contact methods and networking within the Pacific communities. NZ European women were primarily recruited through Web-based methods and special interest Facebook pages.

**Conclusions:**

This cross-sectional study will provide a wealth of data enabling the identification of distinct roles for diet, taste perception, sleep, and physical activity in women with different body fat profiles in modifying the gut microbiome and its impact on obesity and metabolic health. It will advance our understanding of the etiology of obesity and guide future intervention studies involving specific dietary approaches and microbiota-based therapies.

**Trial Registration:**

Australian New Zealand Clinical Trials Registry ACTRN12618000432213; https://www.anzctr.org.au/Trial/Registration/TrialReview.aspx?id=370874

**International Registered Report Identifier (IRRID):**

RR1-10.2196/14529

## Introduction

### Background

Obesity is a global health issue of epidemic proportions [1]. The prevalence in New Zealand (NZ) has increased dramatically over the past three decades, with 1.2 million adults (32% of the population) currently being obese [2,3] and NZ ranking as the third most obese country in the Organization for Economic Co-operation and Development [4]. Obesity is related to significant health inequities, that is, Pacific peoples (69%) and Mãori (50%) are disproportionately affected compared with the general population in NZ (32%), and rates are highest in the most deprived areas [5]. Recent trends in adult obesity in NZ show a significant rise in overweight (currently 64%) and obesity (currently 34%) in women, with major weight gains between the ages of 18 and 45 years [2,4]. This is of significant concern as increased adiposity in women of child-bearing age is associated with acute maternal, neonatal, and ongoing adverse health outcomes, including the perpetuation of increased obesity risk for the next generation [6]. Interventions to halt the epidemic have been unsuccessful [3,4].

Obesity is a complex, multifactorial condition contributing to a chronic prooxidant and proinflammatory state and to deterioration of glucose and lipid metabolism. It increases the risk of several noncommunicable diseases, including type 2 diabetes (T2D), cardiovascular disease (CVD), and some types of cancer [7,8]. Known contributing factors include imbalances in pathways of glucose and lipid metabolism that occur because of variations in quantity and quality of the diet, sedentary lifestyle, and genetic predisposition [9,10]. Obesity arises as a consequence of how the body regulates energy intake, energy expenditure, and energy storage, and it reflects a state of positive energy balance largely caused by westernized environmental pressures [11] resulting in an energy mismatch. This operates through dietary behaviors that do not trigger strong biological opposition [12]. A vicious cycle ensues, involving a state of excessive insulin secretion and a series of metabolic responses that produce systemic insulin resistance [13]. Desensitization to insulin action is accompanied by increased oxidative stress [14] and increased leptin secretion, inflammation, and a decreased ability to metabolize lipid and default energy storage as adipose tissue [15]. Furthermore, changes in the action of endocrine regulators including insulin, leptin, ghrelin, and glucagon-like peptide-1 (GLP-1) disturb appetite regulation in the obese state, rendering sustained weight loss difficult to achieve [16]. In this setting, the regulation of energy balance is biased toward protection against weight loss, further fat accumulation, and disease progression [12,17].

Current public health research to curb obesity is aimed at developing effective food and nutrition policies [11], promoting healthier food choices [18], and community-based interventions [19]. However, the notion that obesity is caused solely because we consume more energy than we expend does not fully explain the substantial increase in obesity [4,20]. Efforts to reduce obesity by inducing a negative energy balance, by counseling people to either eat less or exercise more, are often ineffective because of multiple physiological, behavioral, and social feedback loops [21]. Commonly cited causes of obesity include major changes in our food environment [3,11], which have led to overconsumption of inexpensive, highly palatable, energy-dense, and nutrient-poor foods. These changes in nutritional habits have been reported to influence the gut microbiome, which comprises the bacterial community of the bowel and its associated genetic endowment [4,22]. Current evidence suggests that the gut microbiome plays an important role in the regulation of energy homeostasis and the development of fat storage and obesity [23]. Proposed mechanisms include the microbiome’s capacity to modify energy extraction from food and its ability to influence host signaling pathways that regulate energy metabolism [24]. Furthermore, the gut microbiome can influence satiety and food consumption through gut hormones that trigger endocrine feedback loops regulating appetite [24]. In turn, nondigestible dietary components affect the composition and metabolism of the gut microbiome [25].

The gut microbiome can be viewed as a critical modifiable link between diet and host and may offer new avenues for obesity prevention [26]. Recent studies suggest that people with relatively less diverse microbiomes have higher overall body adiposity and more inflammation-associated characteristics, indicating a higher risk of metabolic diseases [27]. These findings suggest that microbiome complexity and diversity (or richness) may be predictive of the metabolic status of the host and may therefore function as a new biomarker of metabolic health. Dietary patterns that are associated with gut microbiome composition and dietary interventions can increase microbiome richness [25]. In addition, dietary habits may be the most critical factor influencing microbiome status, and therefore, it is critical to understand diet-microbiome interactions and their effect on human health. Finally, a number of other modifiable biological and behavioral factors in the complex causes of obesity appear to be linked with the gut microbiome: sweet and fat taste perceptions influence appetite and dietary behavior and are linked with body weight [28,29], and sleep duration and quality are linked with changes in appetite regulation and energy metabolism [30,31]. Disruption to the circadian biological clock leads to dysbiosis of the gut microbiome [32], and physical activity increases gut microbiome diversity [33].

### Objectives

The PRedictors linking Obesity and the gut MIcrobiomE (PROMISE) study is the first to characterize the gut microbiome in 2 population groups with markedly different metabolic disease risk (Pacific and NZ European women) and different body fat profiles (normal and obese). The potential identification of distinct roles for taste perception, diet, sleep, and physical activity in modifying the gut microbiome and its impact on obesity will greatly advance our understanding of the etiology of obesity and contribute to the discovery of new therapeutic targets. The specific aims of the PROMISE study are to assess in 18- to 45-year-old Pacific and NZ European women: (1) the potential association of gut microbiome complexity and diversity, gene richness, and biochemical endowment in obesity and body fat distribution; (2) interactions between sweet, fat, and bitter taste perception, dietary intake and eating behavior, sleep or physical activity, and their impact on the gut microbiome, metabolic regulation, and body fat profiles; and (3) associations between biomarkers of biological and behavioral risk factors referred to above and specific body fat profiles. This study is designed to test the primary hypothesis that reduced gut microbiome diversity, but high energy-harvest capacity is a key biological driver of obesity and unhealthy body fat distribution in women, whereas greater gut microbiome complexity and gene richness is protective. The secondary hypothesis is that differences in diet, taste perception, sleep, and physical activity affect the associations between the gut microbiome, metabolic regulation, and body fat profiles. This paper reports the outline of the study protocol and the recruitment strategy. Details of the analytical procedures, study outcomes, and clinical measurements will be published elsewhere.

## Methods

### Conceptual Framework and Study Design

**Figure 1:**
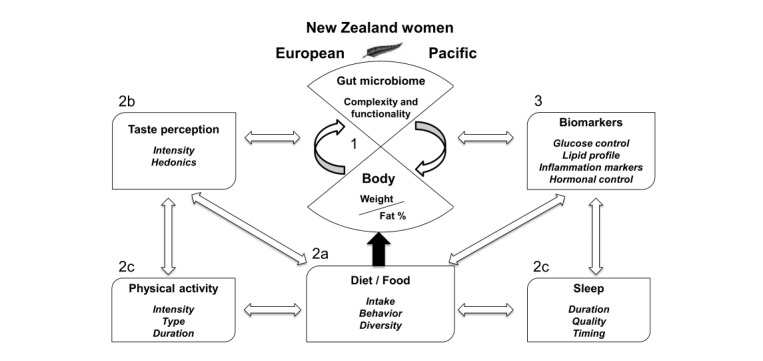
The central role of the gut microbiome in regulating energy homeostasis and body fat distribution.

Comparisons between Pacific and NZ European women, who are known to vary the most in terms of physical, ethnic-cultural, and socioeconomic characteristics in NZ, will allow to assess whether findings are different between groups [2]. A further important distinction between the 2 ethnic groups is that a proportion of the participating Pacific women were born in the Pacific Island of origin, retaining their cultural lineage and dietary traditions, [34] and, as a consequence, may also have a distinctly different gut microbiome. This study involved the assessment of diet, taste perception, sleep, and physical activity, and we investigated the complex interactions between the gut microbiome and its impact on obesity, metabolic markers, and endocrine regulators, as described in Figure 1.

Figure 1 describes the central role of the gut microbiome in regulating energy homeostasis and body fat distribution. Aim 1 of this study investigates differential gut microbiome complexity, gene richness, and functionality between obese body fat profile women and normal body fat profile women stratified by ethnicity. For example, we investigate whether specific bacterial phyla are associated with obesity and/or characterized by relative abundances of genes associated with carbohydrate-active enzymes or whether a highly diverse microbiota with lower energy-harvest capacity is protective. Aim 2a investigates the fundamental influence of diet and food intake on body weight and body fat profile. We assess associations with gut microbiome complexity and functionality and explore whether specific microbiota profiles may be associated with specific dietary intakes or cultural dietary traditions. Aim 2b investigates interactions with sweet, bitter, and fat taste perception and Aim 2c explores the influence of sleep and physical activity. We assess independent effects or effect modification of taste perception, sleep, and physical activity and their impact on the gut microbiome and body fat profiles. Aim 3 examines interactions or effect modification with biomarkers and endocrine regulators and their relationships with food consumption, energy metabolism, and a range of risk factors that shape body fat profiles.

### Participants

**Figure 2:**
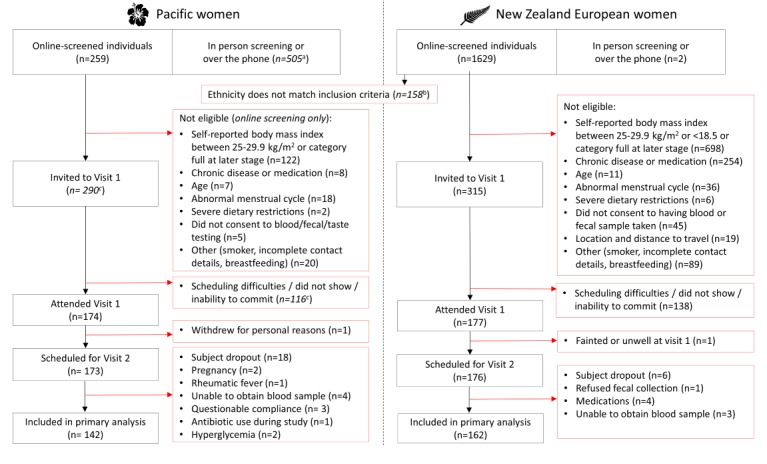
Flowchart describing the recruitment process of the PROMISE study. "a" indicates approximated value for Pacific women in-person screening or over the phone. "b" indicates that ethnicity inclusion was based on Pacific women requiring one parent of full Pacific ethnicity or New Zealand (NZ) European women having lived in NZ for a minimum of 5 years with European parents. "c" indicates approximated values since the majority of study bookings were managed over the phone.

We have conducted a cross-sectional study in 174 Pacific women (who are known to have a high risk of obesity [5]) and 177 NZ European women (who are known to have a moderate risk of obesity [2,5]). Initial screening of body mass index (BMI), based on self-reported weight and height, was conducted on the Web, in person, or via the phone (Figure 2). The 351 participating women, aged 18 to 45 years, were selected such that half in each ethnic group had a normal body fat profile (BMI 18.5-24.9 kg/m^2^) and the other half an obese body fat profile (BMI ≥30.0 kg/m^2^), while recognizing that people with the same BMI can have substantial heterogeneity of body composition and metabolic disease risk factors [35,36].

### Ethics

The study was approved by the Southern Health and Disability Ethics Committee (16/STH/32). This trial was registered at anzctr.org.au (ACTRN12618000432213). All participants were informed in detail about the procedures and measurements and gave written consent. Access to data is restricted to the immediate research team, and only coded data are used for analysis.

### Inclusion and Exclusion Criteria

The inclusion criteria included age 18 to 45 years, being postmenarche and premenopausal (as defined by a regular menstrual cycle for the last year), ethnicity (self-identified Pacific ethnicity and having at least one parent of Pacific ethnicity or self-identified as NZ European ethnicity and having lived in NZ for a minimum of 5 years), written informed consent, willingness to comply with study requirements, and being generally healthy. The exclusion criteria included BMI outside of the predefined normal or obese BMI ranges, pregnant or lactating, presence of any diagnosed chronic illness (eg, T2D and CVD), previous bariatric surgery, severe food allergies, medication that could interfere with appetite or the immune system (eg, appetite suppressants and corticosteroids), current smoker, severe dietary restrictions or avoidances (eg, vegan), and antibiotic use during the last month.

### Participant Recruitment

The Auckland region has a culturally diverse population of 1,534,000, of which approximately 15% are Pacific peoples and 59% NZ European [37]. Health studies sometimes fail to address, or are not appropriately sensitive to, the cultural needs of the participants in terms of the recruitment approach or implementation of the study [38]. We therefore developed a partnership with The Fono, a large Pacific primary health care and social services organization based in Auckland, to integrate culturally appropriate recruitment and research procedures. This included the appointment of a senior Pacific nurse to lead the recruitment and support the data collection and management of the Pacific arm of the study. We also advertised on Pacific Facebook pages and contacted a wide range of special interest and cultural websites; however, we had little uptake from the Pacific organizations or groups that we contacted. As a result, Pacific women were mostly recruited in-person or by phone through the Pacific nurse (see Results). Other recruitment strategies included attending cultural festivals (ie, Pasifika festivals) and other cultural or preorganized events. Another successful strategy was recruiting Pacific students through the University Pacific networks. An additional *Me'a'ofa* (gift or donation) was offered to participants who could successfully enroll another participant who met the inclusion criteria and completed the study, thus enabling recruitment through existing networks. The services of 2 recruitment agencies, PRIME Research Ltd and Consumer Link, were also employed to support the recruitment of Pacific women with a BMI less than or equal to 24.9 kg/m^2^. This was a successful strategy because previous networks were exhausted for this group of participants.

NZ European women typically heard of our study through Web-based advertisements such as Facebook community pages or *public figure* pages (well-known NZ nutritionists, etc), work or university email lists, or other social media sources (see Results). A number of Facebook pages, including local public figure pages, shared details of our study and endorsed the research on our behalf. Community Facebook pages (suburbs around Auckland, local businesses, etc) allowed for wide reach across the Auckland region. In contrast to Pacific women, social media (ie, Facebook) was a highly successful method for NZ European recruitment.

Participant gratuity (NZD $100 gift voucher including options for petrol, shopping center, and supermarket vouchers) was handed out at the end of visit 2 to encourage study completion and the return of all home-use devices and data.

### Study Procedures

**Figure 3:**
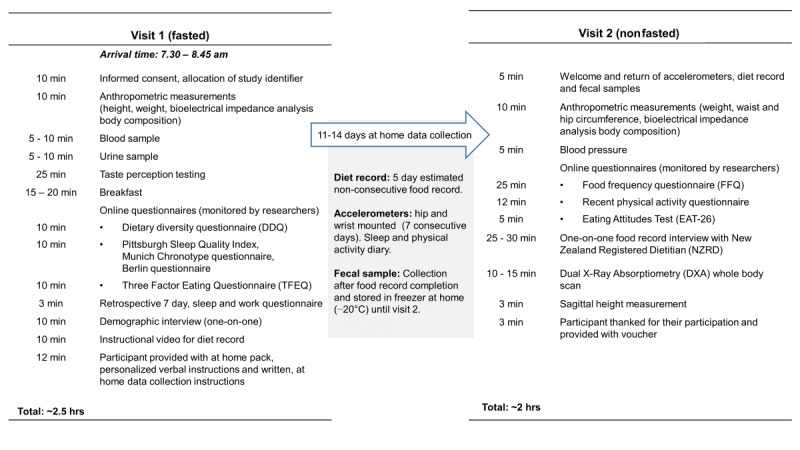
Overview of the PRedictors linking Obesity and gut MIcrobiomE (PROMISE) study visits and schedule.

The PROMISE study was conducted at the Massey University Human Nutrition Research Unit in Auckland, NZ, between July 2016 and September 2017. All eligible participants attended the research unit on 2 occasions, 11 to 14 days apart (Figure 3), where they completed a series of scheduled tasks. Between visits, participants completed the at-home data collection protocols described below. For Pacific women, we ensured that the Pacific nurse was present throughout the duration of Pacific participants’ initial visits. She also performed the phlebotomy procedures for all Pacific participants and addressed all concerns about any aspects of the study that were raised. All Pacific participants were offered door-to-door transport to and from the research unit to enable participation in the study and to reduce the likelihood of *dropouts*.

At visit 1, participants were welcomed, asked to carefully read and sign a consent form, and had the opportunity to ask questions before commencing the study. Each participant was then allocated a unique study identifier (ID). Participants completed a one-on-one interview with a researcher to obtain a range of demographic and health information, including the number of biological children, household and personal income, occupation, work patterns, participants’ birth weight and delivery method (if known), dietary supplement use, frequency of alcohol consumption, and recruitment method.

Figure 3 presents an overview of the PROMISE study visits and the sequence and timing of procedures. Visit 1 included the consent process, blood and urine sampling, taste perception testing, questionnaires, and instructions for at-home data collection. Participants collected data at home for approximately 11 to 14 days. At-home data collection included a 5-day nonconsecutive estimated food record, measurement of sleep and physical activity, and fecal sample collection. Visit 2 included delivery of all at-home collected data, body composition measurements by DXA, questionnaires, and the one-on-one food record interview.

### Blood Samples

Blood samples were obtained between 7:30 am and 9:00 am (after overnight fast, 10-15 hours), by an experienced phlebotomist. A tourniquet was applied moderately to the arm before venipuncture. Blood was drawn into 4 vacutainers (maximum total blood volume was 30 mL), to obtain serum and plasma for analysis of metabolic markers and endocrine regulators. Ethylenediaminetetraacetic acid (EDTA) 10 mL vacutainers (Becton Dickinson) were used for whole blood and stored immediately in a −80° C freezer before the remainder of the sample was placed on ice. The serum vacutainer was left to stand between 30 and 60 min at room temperature (18° C) to clot before centrifugation (10 mL, Becton Dickinson). For endocrine regulators, an additional plasma sample (2 mL Becton Dickinson vacutainer P800 EDTA, aprotinin, and dipeptidyl peptidase IV) was collected. The latter and all other vacutainers were placed on wet ice immediately after collection until centrifugation. Vacutainer tubes were centrifuged at 1500 g for 15 min at 4° C within 1 hour after blood samples were taken. Aliquots of plasma (23 aliquots, 120-500 µL) and serum (6 aliquots, 500-1300 µL) were transferred into prelabeled 1.5 mL microcentrifuge tubes (Eppendorf safe-lock polymerase chain reaction clean tubes) and cryovials (Cryo.S Greiner Bio-One, GmbH) and stored immediately at −80° C. Blood samples were analyzed for a range of biomarkers (eg, plasma glucose, insulin, glycated hemoglobin, lipids, and liver function tests), inflammation markers (eg, high-sensitivity C-reactive protein, interleukin 6 and tumour necrosis factor alpha), and endocrine regulators (eg, GLP-1, ghrelin, leptin, and adiponectin).

### Urine Samples

After completion of blood collection, participants were required to collect a fasting, midstream urine sample. Research staff provided a urine container (BD Vacutainer Urine Collection Cup) labeled with their unique participant ID, cleansing wipes, and gloves. Once urine was collected, participants were asked to place their urine sample container on wet ice inside a clearly labeled polystyrene box before it was further processed by research staff. Midstream urine (14 mL) was pipetted into a prechilled 15 mL falcon tube. Urine samples were centrifuged at 1500 g for 15 min, at 4° C, to remove cellular particles and debris [39]. Ten aliquots of 1000 µL were transferred to each cryovial (Cryo.S, Greiner Bio-One, GmbH) and then transferred to −80° C storage for later metabolomics analysis.

### Anthropometric Measurements

At visit 1, anthropometric measurements included stretched height and fasting weight measurements. All anthropometric measurements were conducted using the International Society for the Advancement of Kinanthropometry (ISAK) protocol [40]. All research staff conducting these measurements were level 1 ISAK trained. BMI was calculated using the Quetelet index (weight/height^2^). At visit 2, waist and hip circumferences were measured with a Lufkin W600PM flexible steel tape with the participant in a relaxed standing position with their arms folded across their chest. Sagittal abdominal diameter was measured at the umbilicus level using the Holtain-Kahn Abdominal Caliper (Holtain Ltd) and assessed in a supine position. Bioelectrical impedance analysis (InBody230) was used to assess body composition at both visits 1 and 2. Body composition measurements were performed using dual-energy x-ray absorptiometry (DXA; Hologic QDR Discovery A, Hologic Inc with APEX V. 3.2 software) at visit 2 to accurately assess body composition profiles in terms of total and regional fat mass. Before DXA scanning, participants were asked to remove their jewelry and if they were pregnant or had a pacemaker or any metal implants. All staff who conducted DXA scanning procedures had Australian and NZ Bone Mineral Society clinical densitometry accreditation.

### Blood Pressure

At visit 2, resting blood pressure was measured with an Omron digital blood pressure monitor (Omron HEM-907, Omron Healthcare Inc) using one of 2 arm cuff sizes (22-32 cm or 32-48 cm). In addition, a record was kept of each participant’s pulse. Three measurements were taken consecutively at 1-min intervals. The mean of the second and third measurements was used to calculate systolic and diastolic blood pressure [41].

### Dietary Intake and Behavior

Dietary intake was assessed for energy, macro- and micronutrient intakes, distribution of food intake throughout the day, and food choice. Our primary method for obtaining current dietary intake data was the gold-standard prospective, 5-day, food record [42-44]. The 5-day estimated food record included 2 weekend days and 3 weekdays. Estimated rather than weighed food records were used to improve adherence and to reduce participant burden [45,46]. Each participant received training for estimating and documenting portions, and every food record was reviewed by an NZ-registered dietitian before a one-on-one, in-depth discussion with the participant to clarify portions of foods consumed, cooking methods, brands of food products reported, and any other ambiguities. Visual portion book aids, household measures (eg, metric cups and spoons), and Web-based tools were used to confirm specific portion sizes and brands consumed, respectively. During these one-on-one interviews, standardized diet behavior-related questions were asked such as intentional meal skipping, snacking behaviors, and food preferences. These detailed sessions were critical to ensure accurate dietary data were captured [47].

It is often debated that all dietary assessment instruments are vulnerable to measurement errors, and significant improvements of dietary intake data quality can be achieved when different dietary intake recording methods are combined, especially combining food recording with food frequency questionnaires (FFQs) [42]. The validated semiquantitative New Zealand Women’s Food Frequency Questionnaire (NZWFFQ) that was adapted from the FFQs used in the National Nutrition Survey NZ [43,44,48,49] was also completed by participants at visit 2. This covered dietary intake retrospectively across the last month, including the days that the 5-day food record was completed, to ensure that the range of actual and usual intakes were captured [42,50]. The 220-item NZWFFQ was administered using a Web-based questionnaire hosted on SurveyMonkey survey software, and live progress was monitored by research staff.

Nutrient analysis of the food record and FFQ data was performed using the Foodworks 9 (Xyris Software Pty Ltd) dietary analysis software, which uses FOODfiles 2016 (developed by the NZ Institute for Plant & Food Research and the NZ Ministry of Health) as a reference food composition table for analysis. In addition, the Xyris database AusFoods 2017 and AusBrands 2017, which are based on the Australian food composition databases AUSNUT 2011-13 (developed by Food Standards Australia New Zealand) were used. The data will be used to assess dietary adequacy in terms of energy and nutrient intakes using the current Australia/New Zealand nutrient reference values [51].

Participants completed the Dietary Diversity Questionnaire to assess food choices and dietary diversity, the Eating Attitudes Test (EAT-26) questionnaire to assess eating disorder risk, and the Three Factor Eating Questionnaire [52] to assess eating behavior in terms of cognitive dietary restraint (restraint), disinhibition of control (disinhibition), and susceptibility to hunger (hunger) by calculating scores for these dimensions and their subcategories.

### Taste Perception

At visit 1, taste perception testing was conducted in a fasting state in individual testing booths at room temperature (20° C), before consuming breakfast. Participants were individually trained on the testing procedure and how to use general labeled magnitude scales [53,54] to rate the intensity and hedonic liking of sweet, bitter, and fat taste stimuli. Four distinct concentrations of glucose (30 g/L, 60 g/L, 120 g/L, and 240 g/L), quinine (0.008 g/L, 0.016 g/L, 0.03 g/L, and 0.06 g/L), and milk fat samples (3.3%, 11.8%, 20.3%, and 37.3% fat) were assessed using water as the control measure. Participants rated each solution by tasting the whole 10 mL of sample. They first rated the water stimuli (control, labeled *sweet*), followed by all sweet taste stimuli, before moving on to bitter taste stimuli and then milk fat stimuli. All stimuli were labeled with a 3-digit number, and the order was randomized within the tastant set. Participants had to rinse their mouth with water between samples. A ranking task was also administered for glucose, quinine, and milk fat samples to evaluate taste sensitivity [55], which was administered at the end of each tastant set. Ranking task stimuli were presented as a set of 4 distinct concentrations, labeled with unique 3-digit numbers to be ranked from the lowest to highest concentration (ie, 30 g/L, 60 g/L, 120 g/L, and 240 g/L).

### Physical Activity and Sleep

To objectively measure sleep and physical activity, participants were fitted with 2 accelerometers during visit 1, 1 hip-mounted w-GT3X accelerometer (Actigraph), to measure physical activity, and a wrist-worn AW2 actiwatch (Phillips Respironics) to measure sleep. Participants were instructed to wear the accelerometers on their hip and nondominant wrist continuously (24-hour protocol) for the following 7 days, except while bathing or participating in water activities such as swimming. During this 7-day collection period, participants recorded in a provided diary the time they woke up each morning and went to sleep each night, as well as any intentional physical activity they engaged in [56]. At visit 2, participants also completed physical activity and sleep questionnaires, including the Recent Physical Activity Questionnaire [57], the Pittsburgh Sleep Quality Index [58], the Berlin questionnaire for sleep apnea [59], and their chronotype was assessed by the Munich Chronotype Questionnaire [60].

### Fecal Samples

Fecal samples were collected by participants at home during the period between visits 1 and 2. At the end of visit 1, participants were briefed on how to collect fecal samples and were provided with a collection kit. This kit contained 2 prelabeled screw-top containers with a scoop in the lid (LBS3805 25 mL, ThermoFisher NZ), 2 larger prelabeled plastic containers (LBS30130 130mL PP, ThermoFisher NZ), kidney dishes, gloves, zip-lock plastic bags, ice-sheets, chiller carry bag, and detailed written instructions. Participants were given an individualized schedule for food record and accelerometer data collection days, with the fecal sample needing to be collected after completion of the food record. Participants collected 2 separate, *walnut-sized* aliquots, from the same fecal sample and were asked to write down the time and date the aliquots were collected. The outer (larger) container was filled with 2 to 3 cm of cold water and the smaller container was placed inside the larger, to create a water jacket. Samples were then placed immediately in their household freezer (−20° C) and transported inside the chiller bag with ice packs when returning to the research unit at visit 2. Similar fecal sample collection methods have been utilized in a range of previous studies [61].

### Gut Microbiome and Bioinformatics

DNA was extracted from fecal samples using methods described in the Human Microbiome Project [62]. In brief, microbial cells in fecal homogenates were physically disrupted by bead-beating, and then DNA was purified using a Mo Bio PowerSoil DNA isolation kit. The DNA was checked for quantity and quality using a combination of gel electrophoresis, nanodrop spectrometry, and Qubit fluorometry. The DNA was shotgun sequenced by NZ Genomics Ltd using Nextera library preparation and pools of 12 barcoded samples run per lane on an Illumina HiSeq 2500 instrument (Illumina).

Gut microbiome sequence data were analyzed using recognized computation (bioinformatics) tools [27]. These tools include the preparation of species-sampling curves that are the classic means of evaluating ecological richness (alpha diversity—biodiversity). As the genomes of all microbial species present in the microbiota were sequenced as small DNA fragments, both phylogenetic (describing what kinds of microbes are there and their relative abundances; MetaPhlAn2, QIIME v2) and functional (the biochemical capacity encoded in the metagenome; HUMAnN2) information are available [61].

### Deprivation Index

Deprivation index was assessed in this study as a measurement of socioeconomic status. New Zealand Deprivation Index 2013 (NZDep2013) combines census data relating to home ownership, housing, qualifications, income, employment, access to transport, communications, and family structure [63]. NZDep2013 provides a deprivation score for each meshblock in NZ. Meshblocks are the smallest geographical area defined by Statistics NZ, with a population of around 60 to 110 people. NZDep2013 groups deprivation scores into deciles, where 1 represents the areas with the least deprived scores and 10 the areas with the most deprived scores. Therefore, a value of 10 indicates that a meshblock is in the most deprived 10% of areas in NZ.

### Power Calculations

We have based our power calculation on previous studies involving metagenome-based measures of gene richness (gene counts) of fecal microbial communities [25,27] where individuals were categorized into clusters of high or low gene counts. Individuals in the low–gene count cluster may be at increased risk of progressing to obesity-associated comorbidities. On the basis of previous studies [27], we assumed the average gut microbiome complexity and gene richness in normal body fat profile women to equate to 640,000 genes. We then assumed a standard deviation of 350,000 in both normal and obese body fat profile women [25,27]. On the basis of these assumptions, we will have 76% power to detect a difference of 25% between both groups (68 per group). We will have 99% power to detect a difference of 40%, as previously observed in a European study [27]. We assumed that 33% of women have *abnormal* taste perception [28,29] or dietary intake [25], and 33% of women with *normal* taste perception and dietary intake have reduced gut microbiome complexity and functionality. On the basis of these assumptions, we will have 97% power to detect a 2-fold difference between both groups. We would have 83% power to detect the same difference if 25% of women with *normal* sweet and fat taste perception or dietary intake had reduced gut microbiome complexity. All power calculations are based on analyses for each ethnic group separately, as differences in associations between ethnic groups may exist.

### Statistical Analyses

Descriptive statistical methods are used to summarize gut microbiome complexity and functionality; dietary intake and behavior; sweet, fat, and bitter taste perception; sleep, physical activity; and biomarkers. Differential gut microbiome complexity and gene richness are analyzed between obese body fat profile women and normal body fat profile women stratified by ethnicity using linear regression analyses. We also assess associations between gut microbiome complexity and functionality and biological and behavioral factors described above. Logistic regression analyses are used to compare reduced versus high gut microbiome complexity and gene richness, based on cut points employed in other studies. We will use multiple regression analysis to assess the independent effects of the biological and behavioral factors and perform stratified analyses to assess effect modification (or interactions). All analyses will be adjusted for potential confounders (socioeconomic position, age, etc).

## Results

### Recruitment of Pacific and New Zealand European Women

The order of recruitment completion of the main groups of study participants was as follows: (1) NZ European BMI 18.5 to 24.9 kg/m^2^; (2) Pacific BMI greater than or equal to 30.0 kg/m^2^; (3) NZ European BMI greater than or equal to 30.0 kg/m^2^; and (4) Pacific BMI 18.5 to 24.9 kg/m^2^. Although the criteria for recruitment of Pacific women generally required that both parents were of Pacific descent, 10% of the Pacific participants recruited for the PROMISE study were accepted if they had only one parent of Pacific descent but identified clearly as being primarily of Pacific ethnicity. The NZ European BMI greater than or equal to 30.0 kg/m^2^ group and the Pacific BMI 18.5 to 24.9 kg/m^2^ group required additional advertising and specifically targeted recruitment strategies in comparison with the other 2 groups of women. The specific recruitment methods and the number of participants recruited through each approach are summarized in Table 1.

Researchers made a number of observations during the study visits that characterized some of the logistical challenges during the recruitment period of the study. For Pacific women, key motivations to participate in the study included, but were not limited to, personal contact with the study facilitators through the Pacific community, *Me'a'ofa* (gift or donation), and interest in the outcomes of the study. For NZ European women, key motivations to participate in the study included, but were not limited to, interest in individual measurements such as blood markers (eg, cholesterol) and body composition measurements (eg, DXA scan), interest in the gut microbiome, *Me'a'ofa* (gift or donation), and interest in the nutrition-related measurements in the study.

### General Characteristics of the Predictors Linking Obesity and the Gut Microbiome Study

A total of 351 participants were eligible to participate in the study (Figure 2). An overview of basic phenotype characteristics of the PROMISE study participants is presented in Table 2. Although our main target was to recruit participants with a normal BMI (18.5-24.9 kg/m^2^) and an obese BMI (≥30.0 kg/m^2^), we also recruited an additional 54 participants in the overweight BMI (25.0-29.9 kg/m^2^) range. The overweight BMI groups were included in this study because, first, some participants had incorrectly assessed their own height and weight before they arrived at the human nutrition research unit, and second, to offset the enormous difficulties of recruiting normal BMI Pacific women.

**Table 1:** Recruitment methods, examples, and the number of participants recruited (feedback from enrolled participants only, N=351).

Recruitment method	Examples	Number recruited^a^	
		Pacific	New Zealand European
The Fono Primary Healthcare Service (West Auckland)	Pacific staff members recruited through their database and wider community. Transport to and from research clinic provided. Radio interview (Radio Samoa 1593 FM; Tongan segment discussing the *gut microbiome*)	102	N/A^b^
Participant word of mouth	University students, previous PROMISE^c^ participants (interest increased with *Me'a'ofa* [gift or donation])	36	21
Community/University Facebook pages/Special interest pages	Auckland (New Zealand) city central and surrounding suburbs, (approximately 38 Facebook pages; with repeat posts) and Pacific Heartbeat	4	52
Public figures Facebook pages^d^	Local nutritionists, sports celebrities	2	26
University/work email lists	Massey University (New Zealand), The University of Auckland (New Zealand), New Zealand Police	3	15
Hospital staff newsletter/magazine	Auckland hospital network and email lists	2	15
Job advertisement site or volunteer page	Job search website, volunteer to participate in research website, student job search website	5	5
One-on-one recruitment (handing out flyers)	University orientation week, early childhood centers, schools	1	9
Websites	University website article, The Fono Primary Healthcare Service	1	3
Magazine articles^e^	National magazines (ie, nutrition, current affairs, and lifestyle). Articles mentioned current study and provided contact details	0	9
Festivals	Local Pacific festivals (ie, Polyfest 2017, Pasifika 2017)	4	1
Posters^e^	Universities, hospitals, health clinics, libraries, cafes, leisure centers, public swimming pools, gyms, community boards, supermarket notice boards	0	11
Recruitment companies	Consumer paid market research databases to recruit Pacific women with a body mass index of 18.5-24.9 kg/m^2^ only	13	N/A
Instagram	Instagram *story* on local nutritionists/public figure Instagram feeds	1	2
Local newspaper articles/website^e^	Local free newspapers	0	3
Internal database of contacts^e^	List of names recontacted who previously completed a similar trial [43] (note: filtered to contact difficult body mass index categories only)	0	3
Neighbourly, Twitter^e^	Local suburb page Web noticeboard, PROMISE study twitter account (@promise_study)	0	2

^a^Level of recruitment based on attendance at visit 1 of the PROMISE study.

^b^N/A: not applicable.

^c^PROMISE: PRedictors linking Obesity and the gut MIcrobiomE.

^d^Public figure pages used at the stage of recruitment where NZ European BMI ≤24.9 kg/m^2^ category was full; eg, one local nutritionist Facebook post resulted in 221 completed screening questionnaires, which resulted in 17 NZ European BMI ≥30.0 kg/m^2^ participants.

^e^Advertisement method successful across NZ European women only.

**Table 2:** General phenotype characteristics and New Zealand Deprivation Index 2013 of the PRedictors linking Obesity and the gut MIcrobiomE (PROMISE) study participants who completed the study protocol (N=304).

Variable		Pacific women (n=142)	New Zealand European women (n=162)
**Normal (BMI^a^ 18.5-24.9 kg/m^2^)**			
	Participants, n	36	79
	Age (years), mean (SD)	24 (6)	30 (6)^b,c^
	BMI (kg/m^2^), mean (SD)	23.0 (1.6)	22.0 (1.5)^b^
	Waist circumference (cm), mean (SD)	74.3 (4.3)	72.5 (4.9)
	Hip circumference (cm), mean (SD)	100.6 (5.6)	97.0 (5.9)^b^
	WHR^d^, mean (SD)	0.74 (0.03)	0.75 (0.05)
	Systolic BP^e^ (mmHg), mean (SD)	109.4 (9.88)	111.7 (10.31)
	Diastolic BP (mmHg), mean (SD)	68.5 (5.86)	70.6 (8.86)
	NZDep2013^f^, mean (SD)	7 (3)	4 (3)^b^
**Overweight (BMI range 25.0-29.9 kg/m^2^)**			
	Participants, n	33	13
	Age (years), mean (SD)	26 (7)	28 (8)
	BMI (kg/m^2^), mean (SD)	27.5 (1.6)	26.4 (1.8)
	Waist circumference (cm), mean (SD)	83.1 (5.1)	79.6 (5.9)
	Hip circumference (cm), mean (SD)	108.6 (4.8)	106.9 (5.8)
	WHR, mean (SD)	0.77 (0.05)	0.75 (0.04)
	Systolic BP (mmHg), mean (SD)	113.1 (7.27)	115.0 (6.88)
	Diastolic BP (mmHg), mean (SD)	70.6 (7.83)	69.5 (3.90)
	NZDep2013, mean (SD)	7 (3)	4 (2)^b^
**Obese (BMI range ≥30.0 kg/m^2^)**			
	Participants, n	73	70
	Age (years), mean (SD)	25 (6)	34 (7)^b^
	BMI (kg/m^2^), mean (SD)	36.9 (5.4)	34.3 (3.0)^b^
	Waist circumference (cm), mean (SD)	100.8 (11.4)	99.3 (8.9)
	Hip circumference (cm), mean (SD)	123.7 (10.9)	121.6 (7.5)
	WHR, mean (SD)	0.81 (0.06)	0.82 (0.06)
	Systolic BP (mmHg), mean (SD)	119.6 (11.5)	121.9 (14.2)
	Diastolic BP (mmHg), mean (SD)	78.9 (9.99)	81.1 (9.39)
	NZDep2013, mean (SD)	8 (2)	5 (2)^b^

^a^BMI: body mass index.

^b^*P*<.01 was deemed statistically significant.

^c^Independent samples *t* tests were performed to determine differences between ethnicities.

^d^WHR: waist-to-hip ratio.

^e^BP: blood pressure.

^f^NZDep2013: NZ Deprivation Index 2013.

## Discussion

### Principal Findings

This paper reports the study protocol and the recruitment strategy of the PROMISE study, the details of the analytical procedures, study outcomes, and clinical and physiological measurements will be published elsewhere. The main objective of the PROMISE study is to characterize the gut microbiome in 2 population groups with markedly different metabolic disease risk (Pacific and European women) and different body fat profiles (normal and obese). The study describes the roles of taste perception, diet, sleep, and physical activity in women with different body fat profiles in modifying the gut microbiome and its impact on obesity and metabolic health. Only healthy participants were included in the study in accordance with strict inclusion and exclusion criteria. We established well-defined protocols of scheduled experimental conditions with standard operating procedures (SOPs) for all domains of the study. All participants followed the same SOPs according to specified timelines, and all specimen samples were treated identically. The main rationale for this approach was to collect high-quality data and to minimize variation related to data acquisition, data analysis, and sampling of biological specimens.

There is a dearth of data in populations at greatest risk of developing obesity. The PROMISE study will help to fill these gaps. Although a cross-sectional design will not infer causality [64], it is a highly efficient approach that will be able to identify distinct roles for diet, taste perception, sleep, and physical activity in modifying the gut microbiome and its impact on obesity and metabolic health. An additional strength of the PROMISE study is the recruitment of women from Pacific and European population groups. It allows us to assess potential differences and commonalities between population groups with markedly different metabolic disease risk profiles and will provide new insights and has the potential to contribute to novel hypotheses. A number of previous studies have presented convincing evidence that the gut microbiome may be a central modifiable link between diet and host and may be likely to offer new avenues to tackle obesity [22,25,26]. However, many important questions remain. First, how important is the ratio of the bacterial phyla *Firmicutes* to *Bacteroidetes*? Highly publicized studies reported low abundances of *Bacteroidetes* and higher abundances of *Firmicutes* as a characteristic of obesity [65]. However, later studies failed to find this association and, in general, meta-analyses show that most obesity-microbiome studies in humans to date have been underpowered to determine valid differences between groups [66]. In addition, there is increasing evidence that whole genome shotgun sequencing, as used in the PROMISE study, has multiple advantages compared with the 16S amplicon method used in many previous studies. The advantages of shotgun metagenomics sequencing include enhanced detection of bacterial species, increased detection of diversity, and increased prediction of the most dominant gene pathways that are present in the particular genes [67]. Furthermore, much of the previous work has been conducted in mice, and the few human studies available were small and need to be replicated in larger studies. Second, how strong is the association between low gene count clusters and obesity? Metagenomic measures of gene richness (gene counts) of gut microbial communities have categorized individuals into clusters of high or low gene count, but the exact nature of this relationship is not known [25,27]. Third, is there an association between relative abundances of bacterial species with obese or lean phenotypes in humans [68]? Fourth, is there an association between abundance of specific gut microbiota with dietary intake of particular food groups or dietary patterns [69,70]? Finally, do ethnic and/or cultural differences, sleep, and physical activity modify associations between the gut microbiome and obesity?

Given the cultural diversity of the participants, it was vital that research staff from the Pacific community were actively involved in performing the study to ensure the study was conducted in a culturally appropriate manner and to support the collection of quality data and successful outcomes [71]. Furthermore, there is convincing evidence that community-based participatory research approaches, involving community members and organizational representatives can overcome recruitment challenges and enhance the quality of a study [71-73]. The participation of a senior Pacific research nurse in the research team and the partnership with The Fono were invaluable in providing support and understanding from a participant perspective. In NZ, the essence of showing respect and kindness is described as *manaakitanga*, which encompasses hosting visitors with care, developing a nurturing relationship, and being a responsible host [74]. Adoption of *manaakitanga* within the framework of the PROMISE study, incorporating socially and emotionally grounded beliefs, enhanced participant engagement in what could commonly be perceived as a formal clinical research setting. Therefore, we recommend that future studies incorporate a range of strategies and culturally appropriate approaches to support community-based engagement throughout all aspects of the research [38].

### Strengths and Limitations

A remarkable experience of the recruitment process was the success of using social media as a recruitment tool for NZ European women. Overall, we found that employing multiple recruitment methods, including social media (eg, community Facebook pages), newspaper advertisements, and business circulars (eg, work place email lists) gave this study a wider representation of the general population. It is important to keep in mind that although Auckland city has the highest population of resident Pacific peoples, the total number of Pacific women living in Auckland is much lower than that of NZ European women. In addition, many Pacific communities live in regions that are over 25 km away from the research unit such that transport issues were a significant barrier for some Pacific participants. Therefore, it was not surprising that Pacific women were more challenging to recruit than NZ European women. It has been recognized that barriers to participating in clinical research include fear and lack of trust in the study procedures or in research staff. In contrast, motivating factors include free health care access, feeling connected to the research outcomes that may support family or friends in the future (eg, developing treatments for specific diseases), and monetary incentives [72]. The PROMISE study team embraced these critical factors, paid attention to using appropriate language, and generated a culturally and gender-appropriate setting during all procedures (ie, DXA scanning) to contribute to a positive experience for participants [72].

The recruitment of participants faced a number of challenges. For example, women with a high BMI may have felt less motivated to take part in obesity-related research because of a concern of negative evaluation, as reported previously [75]. Furthermore, difficulties in recruiting Pacific women with a normal BMI in this study were because of their low number within the general Pacific population, only 8% of adult Pacific women have a BMI between 18.5 and 24.9 kg/m^2^ [5,37]. To address these barriers, additional efforts were made to advertise and recruit Pacific women into the PROMISE study. Therefore, we extended the recruitment range, especially for Pacific women, to include the overweight BMI range (25.0-29.9 kg/m^2^). Furthermore, we increased the *Me'a'ofa* and provided transport to and from the research unit to encourage participation and completion of the study protocol. The disadvantage of such tailored recruitment approaches is the risk of increasing selection bias. Although we are not able to quantify the potential selection bias because of the differences in the success rate of the range of recruitment methods presented in Table 1, we have made every effort to ensure participants are representative of each population group [76]. Furthermore, we tailored our advertising and recruitment strategy in a way that was most culturally appropriate for each population group; it ensured participant engagement and motivation, which is known to enhance data quality and study completion [38,77].

Challenges of a cross-sectional study design include the temporality of single assessments and the potential bidirectional nature of some associations. Furthermore, multiple comparisons and a large number of assessments and outcome variables and the potential for complex interactions may require further stratification. However, the study design and recruitment emphasis on obese *versus* normal BMI categories in the PROMISE study is an efficient approach to identify and contrast biological parameters that are associated with obesity-related metabolic disease risk. Most previous studies have focused on only 1 or 2 aspects that may influence the gut microbiome and obesity. The comprehensiveness of the PROMISE study design and our multidisciplinary approach are a particular strength. It will greatly advance our understanding of the etiology of obesity and will guide future longitudinal studies and interventions involving specific microbiota-based therapies, linking the outcomes of our study with strategies for the design of foods that offer metabolic health benefits through changes of the gut microbiome.

## Abbreviations

BMI: body mass index
BP: blood pressure
CVD: cardiovascular disease
DXA: dual-energy x-ray absorptiometry
EDTA: ethylenediaminetetraacetic acid
FFQ: food frequency questionnaire
GLP-1: glucagon-like peptide-1
ISAK: International Society for the Advancement of Kinanthropometry
NZ: New Zealand
NZDep2013: New Zealand Deprivation Index 2013
NZWFFQ: New Zealand Women’s Food Frequency Questionnaire
PROMISE: PRedictors linking Obesity and gut MIcrobiomE
SOP: standard operating procedure
T2D: type 2 diabetes
WHR: waist-to-hip ratio
